# Update on the Treatment of Diabetic Retinopathy

**DOI:** 10.1100/tsw.2008.25

**Published:** 2008-02-06

**Authors:** Jennifer L. Wilkinson-Berka, Antonia G. Miller

**Affiliations:** Department of Immunology, Monash University, Alfred and Medical Research and Education Precinct (AMREP), Commercial Road, Prahran, Victoria, 3004, Australia

**Keywords:** diabetic retinopathy, angiotensin, VEGF, corticosteroids, protein kinase C, advanced glycation end-products, growth hormone

## Abstract

Retinopathy is the most feared complication of diabetes, compromising quality of life in most sufferers. Almost all patients with type 1 diabetes will develop retinopathy over a 15- to 20-year period, and approximately 20–30% will advance to the blinding stage of the disease[1]. Greater than 60% of patients with type 2 diabetes will have retinopathy. This situation is highlighted by the frightening statistic that diabetic retinopathy (DR) remains the most common cause of vision impairment in people of working age in Western society. With the global epidemic of type 2 diabetes, this predicament is set to worsen as over 360 million people are projected to suffer from diabetes and its complications by 2030. Vision loss from diabetes is due to a number of factors, including haemorrhage from new and poorly formed blood vessels, retinal detachment due to contraction of deposited fibrous tissue, and neovascular glaucoma resulting in an increase in intraocular pressure. Diabetic macular oedema is now the principal cause of vision loss in diabetes and involves leakage from a disrupted blood-retinal barrier. In terms of treatment, there is clear evidence that strict metabolic and blood pressure control can lower the risk of developing DR and reduce disease progression. Laser photocoagulation and vitrectomy are effective in preventing severe vision loss in DR, particularly in the most advanced stages of the disease. However, both procedures have limitations. This review examines evidence from preclinical and clinical studies that shows that targeting inhibition of the renin-angiotensin system, vascular endothelial growth factor, corticosteroids, protein kinase C, growth hormone, and advanced glycation end-products are potential treatments for DR.

## STAGES AND PATHOGENESIS OF DIABETIC RETINOPATHY

Diabetic retinopathy (DR) is generally classified into nonproliferative diabetic retinopathy (NPDR), which comprises the early stages of the disease, and proliferative diabetic retinopathy (PDR), which is the most serious and vision-threatening stage. In 2002, the Global Diabetes Retinopathy Group reclassified the stages of severity of DR such that five scales now exist based on increasing risks of vascular lesions[2]. NPDR has four scales, with the first level defined as "no apparent retinopathy". The second level is known as "mild NPDR" and includes the appearance of microaneurysms. The third level is "moderate NPDR" and is defined as more than microaneurysms, but less than severe NPDR. The fourth level, "severe NPDR", includes the appearance of 20 intraretinal haemorrhages in each of the four retinal quadrants, venous beading in two or more quadrants, and prominent intraretinal microvascular abnormalities in one or more quadrants. PDR is the fifth level and is defined as "definite neovascularisation or vitreous/preretinal haemorrhage". Diabetic macula oedema (DMO) is classified into three stages. The first stage is "no apparent retinal thickening or lipid in the macula". The second stage is "some apparent retinal thickening or hard exudates", while the third and most serious stage has three subdivisions of "mild", "moderate", or "severe", which depend on the extent of retinal thickening and the location of hard exudates relative to the fovea. Severe DMO involves the appearance of hard exudates in the centre of the macula.

The earliest stage, NPDR, features thickening of capillary basement membrane, apoptosis, or "drop out" of pericytes, microaneurysms, intraretinal haemorrhages, and cotton-wool spots[3,4,5,6]. The gradual closure of retinal vessels results in localised areas of tissue ischaemia giving rise to venous beading and intraretinal microvascular abnormalities, increasing retinal haemorrhage and exudation. Tissue ischaemia may also be worsened by endothelial cell apoptosis, resulting in acellular capillaries (devoid of both pericytes and endothelial cells). Adherent leukocytes can also contribute to vessel closure. The advancement to PDR is viewed to be a consequence of tissue ischaemia and the subsequent up-regulation of angiogenic growth factors, which stimulate new blood vessel growth over the optic disc or elsewhere in the retina (Fig. 1). These new vessels can advance into the vitreous cavity, leading to haemorrhage (Fig. 1) and tractional retinal detachments due to the contraction of associated fibrous tissue. Neovascularisation and fibrosis can develop in the iris, leading to rubeosis iridis, which affects the outflow of aqueous humour, resulting in neovascular glaucoma and an elevation in intraocular pressure, which compromises vision.

## TREATMENT OF DIABETIC RETINOPATHY

### Laser Photocoagulation and Vitrectomy

Laser and surgical interventions that were developed in the 1950s and 1960s remain the current treatment for DR and are predominately targeted at the most serious stages of the disease[7,8]. Laser photocoagulation, in which laser burns are applied over the retina, removes neovascular vessels and also areas of tissue ischaemia in an attempt to prevent further stimulation of angiogenic growth factors. The Diabetic Retinopathy Study (DRS)[9] and Early Treatment Diabetic Retinopathy Study (ETDRS)[10] have provided evidence that photocoagulation can reduce vision loss, with panretinal photocoagulation lowering by approximately 50% the risk of severe vision loss, and focal and grid laser photocoagulation limiting vascular leakage in clinical significant DMO[11]. In some people with less severe PDR, laser photocoagulation may be warranted under particular circumstances, such as patients with type 2 diabetes, a history of poor patient follow-up, and the presence of concurrent risk factors that suggest rapidly progressing retinal disease[12]. Vitrectomy is also used for the treatment of advanced DR. Vitrectomy is associated with increased visual acuity and is usually aimed at removing nonclearing haemorrhage or fibrosis, areas of tractional retinal detachment, active progressive PDR, and DMO[13,14].

Although laser photocoagulation and vitrectomy significantly reduce the risk of severe vision loss in diabetic patients, there remain inherent limitations. Such procedures are usually applied when the disease is advanced and the retina seriously damaged, and therefore do not target early pathology and disease progression. Moreover, these procedures are associated with adverse events. For photocoagulation, this includes losses in colour vision, night blindness, and the advancement of DMO and tractional detachment[15,16]. Vitrectomy can be accompanied by recurrent vitreous haemorrhage, premature development of cataract, rubeosis iridis, retinal detachment, and retinal tear[17].

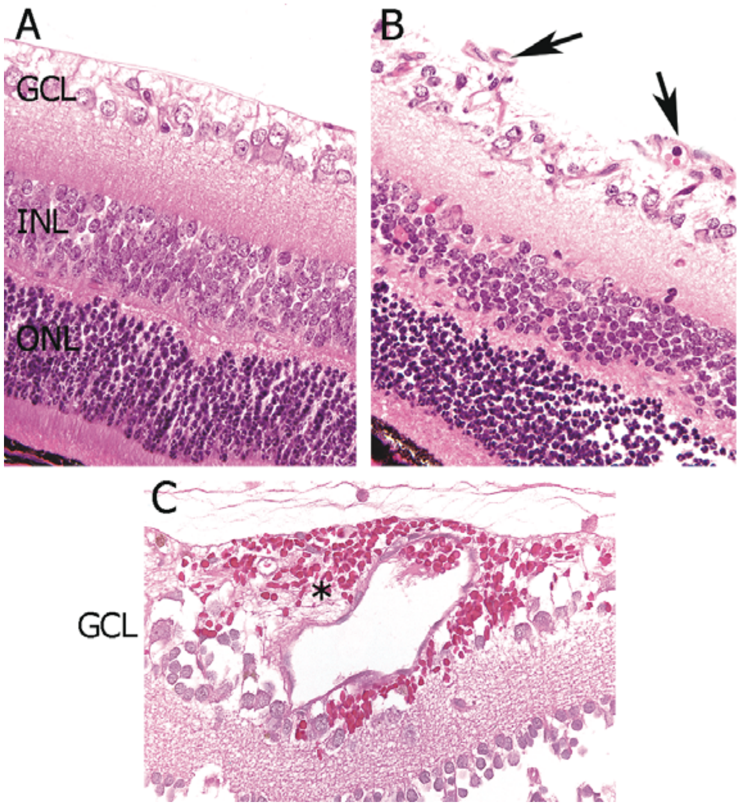

**FIGURE 1**. Four-micron paraffin sections of retina stained with haematoxylin and eosin. GCL, ganglion cell layer; INL, inner nuclear layer; ONL, outer nuclear layer. (A) Normal retina, (B) PDR with blood vessels (arrows) extending into the vitreous cavity, (C) PDR showing intraretinal haemorrhage (asterisk).

## Systemic Factors

There is a wealth of evidence from clinical research to indicate that the control of systemic factors, such as blood glucose, blood pressure, and lipids (Table 1, Fig. 2), has significant benefits for the treatment and progression of DR.

### *Glycaemic Control*

Studies such as the Diabetes Control and Complication Trial (DCCT) in type 1 diabetes (T1D) and the United Kingdom Prospective Diabetes Study (UKPDS) in type 2 diabetes (T2D) have clearly shown that intensive control of blood glucose reduces the risk of developing the complications of diabetes, including retinopathy[18,19]. The DCCT comprised 1,441 T1D patients in 29 centres who were studied between 1983 and 1993[18]. Patients had either no retinopathy or very mild to moderate NPDR. The DCCT reported that during the average treatment period of 6.5 years, the risk of developing DR was substantially lower in the intensive treatment group, where blood glucose was kept to as normal levels as possible with three or more daily insulin injections or treatment with an insulin pump, compared to the conventionally treated group who had one to two insulin injections each day[18]. Many of the DCCT patients also participated in the Epidemiology of Diabetes Interventions and Complications (EDIC) study[20]. A major aim of the EDIC was to determine if the benefits achieved in the DCCT with intensive insulin therapy persisted[20]. The EDIC reported that even 4 years after the end of the DCCT, the risk of progressive

**TABLE 1**
**Examples of Current Drug Targets and Treatments for DR**

| Targets | Treatments |
|---|---|
| Lipids | Fibrates<br>Statins |
| Renin-angiotensin system | Angiotensin type 1 receptor blockade |
| VEGF-A | Pegatanib (Macugen)<br>Ranizumab (Lucentis)<br>Bevacizumab (Avastin)<br>VEGF Trap |
| VEGF-R1 | Sirna-027 |
| Corticosteroids | Trimacinolone acetonide<br>Fluocinolone acetone |
| Protein kinase C | Ruboxistaurin |
| Advanced glycation end-products | Aminoguanidine (Pimagedine) |
| Aldose reductase | Aldose reductase inhibitors (ARI-809) |
| Vitamin E | |
| Nicotinic acetylcholine receptor | ATG3 |
| PEDF | AdPEDF |

VEGF, vascular endothelial growth factor; VEGF-R1, vascular endothelial growth factor receptor-1; PEDF, pigment epithelial derived growth factor; AGT-3 (mecamylamine), nictotinic acetylcholine receptor antagonist; AdPEDF, adenoviral pigment epithelial derived growth factor.

retinopathy and nephropathy remained reduced despite a return to rising hyperglycaemia[21]. Additional analysis of data from the DCCT has shown that although glycaemic variability may occur in T1D patients with similar mean blood glucose levels, glycaemic variability is not a risk factor for DR[22]. Overall, these studies indicate that the implementation of intensive therapy as early as possible leads to some protection against the future risk of progressive retinopathy. However, there are reports that intensive glycaemic control can have some adverse effects, including a worsening of DR that may be attributable to a rapid reduction of glycated haemoglobin ($HbA_{1c}$), hypoglycaemic episodes, ketoacidosis, and weight gain[23,24,25].

## Lipid Control

The dyslipidaemia of T2D is characterised as comprising a lipid triad of low density lipoprotein (LD)-cholesterol (LDL-C), elevated triglycerides, and the presence of small dense LDL[26]. LDL in diabetes is belied by the presence of increased small dense LDL and elevated apoliprotein B (apoB). There is observational evidence that elevated serum lipids increase the risk of DR, and this is particularly relevant to the development of DMO. For instance, in a subgroup of the HOORN study, which included 626 patients, plasma total and LDL-C levels showed associations with hard exudates in DR[27]. In the Wisconsin Epidemiologic Study of Diabetic Retinopathy (WESDR), the presence of hard exudates was significantly associated with increased serum cholesterol levels amongst insulin-using patients[28]. Similar findings were reported in the ETDRS[29]. These results suggested that lipid-lowering therapies may be of benefit in the reduction of hard exudates and the association vision loss in patients with DR.

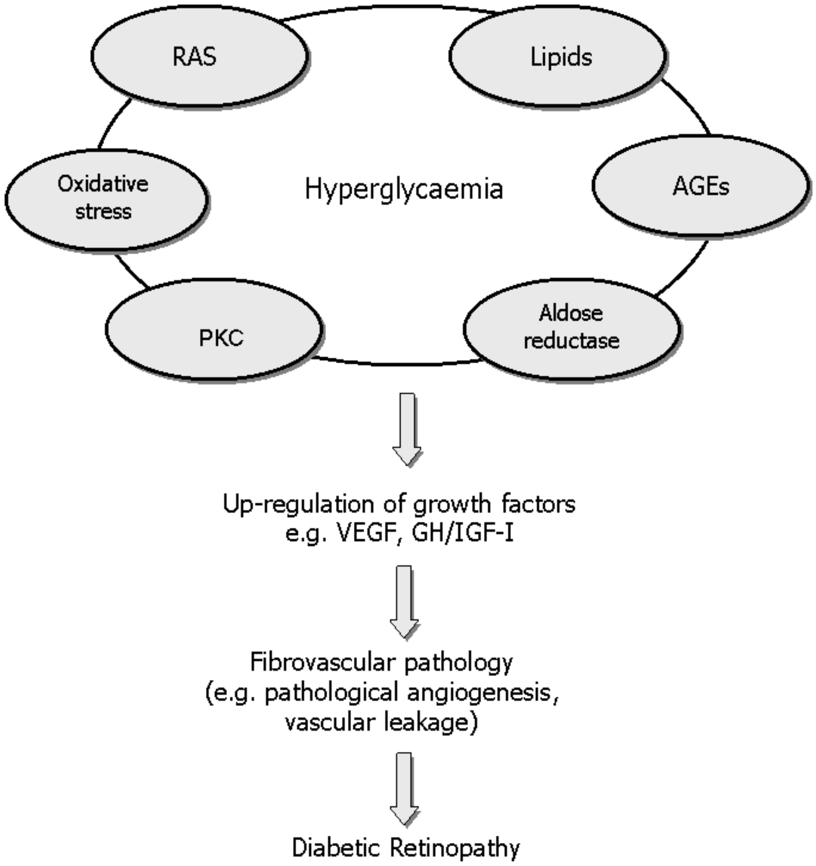

**FIGURE 2**. Schema summarising the factors involved in the pathogenesis of DR. RAS, renin-angiotensin system; AGEs, advanced glycation end-products; PKC, protein kinase C; VEGF, vascular endothelial growth factor; GH, growth hormone; IGF-I, insulin-like growth factor-I.

## Fibrates

Fibrates are agents which activate a series of genes that affect lipid metabolism by binding to the peroxisome proliferator-activated receptor-α (PPAR-α). This ligand receptor complex acts as a copromoter or repressor in the production of apolipoproteins, lipases, lipid-oxidasing enzymes, and transmembrane lipid transport systems. Fibrates are known to increase high density lipoprotein (HDL) cholesterol, reduce triglycerides, and increase particle sizes of both LDL and HDL. An early study with clofibrate reported a reduction in hard exudates, but no improvement in visual acuity in patients with clinically significant DMO[30]. The Fenobirate Intervention and Event Lowering in Diabetes (FIELD) study recruited a low-risk population with a lipid profile that would be more usually treated with a statin. FIELD comprised amongst 9,795 patients with T2D studied in Australia, New Zealand, and Finland with a median follow-up of 5 years. FIELD reported a reduced rate of total cardiovascular events in individuals treated with fenofibrate. In terms of retinopathy, fenofibrate patients were less likely to need laser photocoagulation (1.6%); however, the severity of the DR and the type of laser treatment were not reported[31].

## *Statins*

3-Hydroxy-3-methylglutaryl coenzyme A (HMG-CoA) reductase inhibitors, or statins, inhibit the generation of mevalonate, a precursor of cholesterol and associated products. Statins are commonly used to lower cholesterol and have been recently shown to reduce the risk for cardiovascular events in diabetic patients with or without coronary artery disease. Statins reduce serum LDL-C and apoB levels, but have little effect on particle size. In terms of diabetic microvascular disease, statins have reported benefits[32,33,34,35]; however, their efficacy for DR has not been thoroughly investigated. To date, most clinical studies have used small sample sizes, but have shown reductions in the progression of DR[36], the appearance of hard exudates and microaneurysms[37,38], and retinal blood flow velocities[39]. The Collaborative Atorvastatin Diabetes Study (CARDS), a randomised controlled trial of 2,830 patients with T2D, did not find atorvastatin to be effective in reducing DR progression[40]. A substudy of the ongoing trial, The Atorvastatin Study for Prevention of Coronary Heart Disease Endpoints in Non-Insulin-Dependent Diabetes Mellitus (ASPEN), will also evaluate the effects of atorvastatin on DR[41].

## Blood Pressure Control

There is considerable evidence that hypertension is a risk factor for DR[42]. The effects of blood pressure–lowering agents on the progression of DR have been studied in a number of randomised controlled trials, particularly in patients with T2D. The UKPDS trial reported that people with diabetes who also have hypertension are more likely to experience a progression in their DR[43]. The UKPDS comprised 1,148 hypertensive patients with T2D allocated to tight control of blood pressure (<150/85 mmHg) and 390 patients to less tight blood pressure control (<180/105 mmHg)[43]. Antihypertensive therapy consisted of either the angiotensin converting enzyme (ACE) inhibitor captopril or the β-blocker atenolol. After 9 years of follow-up, tight blood pressure control was associated with a 34% reduction in the rate of progression of DR by two or more steps using the modified ETDRS scale. In addition, there was a 47% reduction in the deterioration of visual acuity by three lines using the ETDRS chart[43]. In contrast, the Appropriate Blood Pressure Control in Diabetes (ABCD) trial[44] reported no difference in the progression of DR in hypertensive T2D patients who had intensive (132/78 mmHg) or moderate (138/86) blood pressure control. In this trial, 470 patients were randomised to receive the calcium channel blocker nisoldipine or the ACE inhibitor enalapril, and the follow-up period was 5 years.

Recently, the Action in Diabetes and Vascular Disease Controlled Evaluation (ADVANCE) trial reported the findings of a study of 11,140 T2D patients performed by 215 collaborating centres in 20 countries[45]. Patients were randomised to receive either a fixed combination of the ACE inhibitor perindopril, plus the diuretic indapamide, or placebo. Fixed combination therapy was given in addition to current therapy. However, if current therapy comprised an ACE inhibitor other than perindopril, it was withdrawn and patients were offered open-label perindopril. After a mean follow-up period of 4.3 years, those assigned fixed therapy had a mean reduction in systolic blood pressure of 5.6 mmHg and diastoiic blood pressure of 2.2 mmHg. Fixed combination therapy was associated with a reduction in the risk of major vascular events, including death. However, there was no effect of perindopril plus indapamide on the incidence of new or worsening microvascular eye disease, including that defined by retinal photocoagulation. Further and perhaps more sensitive information will be obtained from analyses of retinal photographs obtained in a subgroup of patients in ADVANCE known as the ADVANCE Retinal Measurements (AdRem) study[46].

Fewer studies have evaluated the effects of antihypertensive therapy in T1D. The EURODIAB Controlled trial of Lisinopril in Insulin-dependent Diabetes (EUCLID) evaluated the effect of the ACE inhibitor lisinopril on the progression of DR in normotensive normoalbuminuric patients. After 2 years, lisinopril reduced the progression of DR by 50% and progression to PDR by 80%. The studies limits include a short follow-up period of 2 years and differences in baseline glycaemic levels between groups[47]. The findings of EUCLID are consistent with a small study of 20 normotensive T1D patients that showed a protective effect of the ACE captopril on fluorescein leakage through the blood-retinal barrier[48]. A large randomised clinical trial is currently ongoing in T1D. The DR Candesartan Trials (DIRECT) programme has been established to determine whether angiotensin type 1 receptor blockade with candesartan can prevent the incidence and progression of DR[49]. DIRECT involves over 5,000 patients recruited from about 300 centres worldwide, with patients being either normotensive or treated hypertensive individuals. The results of DIRECT are anticipated in 2008.

There is also evidence that the presence of retinopathy in nondiabetic people may predict the subsequent development of diabetes and hypertension, independent of other risk factors[50]. In a 15-year follow-up study from the Beaver Dam Eye Study, Klein and colleagues evaluated the presence of retinopathy in 3,402 people who at baseline examination were without diabetes and aged 43–86 years[50]. Retinopathy in nondiabetic people was associated with the incidence of hypertension. Furthermore, retinopathy and particularly the presence of dot haemorrhages in younger nondiabetic people (less than 65 years of age) were associated with the increased incidence of diabetes. Other clinical studies using digitised imaging of retinal photographs have shown that signs of focal microvascular abnormalities (arteriolar narrowing, venular dilation, and arteriovenous nicking) are associated with the development of hypertension and diabetes[51,52,53] and the progression of DR[54].

### *Angiotensin II*

Although lowering blood pressuring is clearly beneficial for the treatment of DR, there is still little information about how hypertension contributes to DR and which antihypertensive medication is most efficacious. A rather strong case can be made for targeting angiotensin II. The renin-angiotensin system is disturbed in diabetes patients (reviewed [55]) and in patients with PDR; prorenin, the inactive precursor of renin, is elevated in plasma and vitreous fluid compared to nondiabetic subjects[56,57,58]. All components of the renin-angiotensin system (RAS) have been identified in the retina, including renin, ACE, ACE2, and the angiotensin type 1 and angiotensin type 2 receptors[59,60,61,62,63,64,65]. Their location in blood vessels, neurons, and glia suggests that the retinal RAS may influence not only vascular events, but also retinal function. There is evidence that this is the case, with experimental studies indicating that ACE inhibition and angiotensin type 1 receptor blockade inhibits the increase in retinal VEGF and VEGF receptor 2 (VEGFR-2), vascular leakage, inflammation, basement membrane thickening, acellular capillaries, and losses in retinal function (electroretinogram) that occurs in diabetes[66,67,68,69,70,71,72]. Recent evidence suggests that angiotensin II blockade, rather than antihypertensive treatment per se (β-blockade), is retinoprotective in experimental DR[66,67,73]. Other RAS peptides may also be involved in organ pathology. For example, the recent evidence that the N-terminal degradation product angiotensin IV influences cell growth and inflammation in cardiovascular tissues[74] has implications for diabetic complications, including DR.

## Biochemical Pathways and Growth Factors

A number of factors affecting cellular function, growth, and survival are implicated in the pathogenesis of DR. The factors highlighted in this section were chosen on the basis of the most compelling evidence from preclinical and clinical studies for their causative role in DR. Other factors with emerging roles in DR are presented in the final section of the Review, titled “Other Potential Treatments”. This information is summarised in Table 1 and Fig. 2.

### *Protein Kinase C Inhibitors*

Hyperglycaemia-induced metabolic factors are viewed to contribute to diabetic complications, including retinopathy. Protein kinase C (PKC) is a family of serine/threonine kinases that consists of 12 isoforms.

These isoforms are classified according to whether they contain domains that bind $Ca^{2+}$ or diacylglycerol, both of which regulate the kinase activity. Various PKC isoforms are changed in response to hyperglycaemia and each may mediate unique functions[75]. These functions may depend on the cellular distribution of each isoform in tissues and binding to specific anchoring proteins after activation and translocation[75]. The specific effects of the PKC isoforms are being revealed with the advent of isoform-specific PKC inhibitors. The highly selective PKCβ inhibitor ruboxistaurin mesylate has been most extensively studied in diabetic complications. Ruboxistaurin improves diabetic nephropathy in experimental models, which includes a reduction in glomerulosclerosis, mesangial expansion, and fibrosis in the presence of hypertension[76,77]. These results have led to clinical trials evaluating the effects of ruboxistaurin on diabetic complications. In a phase II, randomised, multicentre, clinical trial of 123 T2D patients, ruboxistaurin improved albuminuria, and maintained glomerular filtration rate and proteinuria in diabetic patients who were already treated with angiotensin II blockers[78] .

In the retina, PKC activity is increased after 2 months of experimental diabetes and can be attenuated with ruboxistaurin[79]. In terms of retinal pathology, VEGF's induction of vascular leakage is associated with increased PKC and ruboxistaurin can reduce vasopermeabiility[80]. Ruboxistaurin has also has been reported to attenuate the increase in leukocyte entrapment in streptozotocin-diabetic rats[81]. In an 18-month trial, 41 patients with DMO were randomly assigned to receive 4, 16, or 32 mg/day ruboxistaurin or placebo. Retinal vascular leakage was assessed using vitreous flurometry at baseline and after 3, 12, and 18 months. Ruboxistaurin reduced retinal vascular leakage by 30% compared with placebo[82]. The Protein Kinase C Diabetic Retinopathy Study (PKC-DRS) randomised 252 patients with moderate to severe NPDR to receive either ruboxistaurin (8, 16, or 32 mg/day) or placebo for 36–48 months. No significant differences in DR progression were observed, although patients with definite DMO at baseline and treated with the highest dose of ruboxistaurin had a significant reduction in the risk of moderate visual loss[83]. Treatment was well tolerated with only a few adverse effects[83]. A larger study, PKC-DRS2, which randomised 685 patients at 70 clinical sites, showed similar results[84]. The PKC-DME study was a multicentre, double-masked, randomised, placebo-controlled study of 686 patients receiving placebo or ruboxistaurin orally (4, 16, or 32 mg/day) for 30 months[85]. Patients with macular oedema had mild to moderate PDR and no prior laser treatment. PKC-DME reported no significant reduction in progression of DR or incidence of macular oedema. There was, however, a trend toward a reduction in clinically significant DMO in patients treated with 32 mg ruboxistaurin[85]. Another PKC inhibitor, PKC412, has been trailed in a randomised, multicentre, double-masked, parallel-group study in which 141 subjects received placebo or PKC412 (50, 100, or 150 mg/day) for up to 3 months[86]. The highest doses were associated with a decrease in retinal thickening and a small, but significant ($p$ = 0.007), improvement in visual acuity at 3 months compared with baseline. Side effects include diarrhoea, nausea, and vomiting[86]. Ongoing trials are evaluating the effects of ruboxistaurin on clinically significant macular oedema over an 18-month period[87].

## *Advanced Glycation End-Products*

Glycation (nonenzymatic glycosylation) processes, also known as the Maillard reactions, result in a series of modifications on long-living matrix structural proteins, such as type IV collagen, laminin, and fibronectin[88]. These modifications are formed as a result of carbohydrate reactions with free amino groups resident within these proteins and are generally termed advanced glycation end-products (AGEs). In humans, AGEs are mainly detected by quantitation of pentosidine and N-carboxy-methyl lysine (CML). The biological effects of AGEs are mediated in part by the specific cell surface receptors, which include the receptor for advanced glycation end-products (RAGE), galectin-3, CD36, and the macrophage scavenger receptor. It has been suggested that the interaction of AGEs with their receptors activates downstream signalling to initiate a wide range of abnormal responses in cells and tissues, such as the inappropriate expression of growth factors, alterations in cell growth, accumulation of extracellular matrix, and initiation of cell death[89].

AGE accumulation occurs as a consequence of ageing and also hyperglycaemia due to long-term exposure to elevated glucose concentrations[90]. In DR, AGE formation has been reported in retinal vessels of animals[91] and in people, high serum levels of CML have been associated with advanced stages of retinopathy in T2D[92]. In addition, AGE[93], RAGE, and its ligands[94] are all increased in the vitreous cavity of people with DR. Preclinical models have been used to establish a pathogenic role for AGEs in DR, with AGE administration inducing vascular leakage, leukostasis, and the up-regulation of profibrotic growth factors[95]. Inhibitors of AGEs are retinoprotective, reducing blood-retinal barrier breakdown, leukostasis, retinal capillary basement membrane thickening, acellular capillaries, and expression of inflammatory mediators and extracellular matrix factors[96,97,98,99,100,101]. However, the AGE inhibitor aminoguanidine did not improve losses in retinal function (electroretinogram) in diabetic rats[71]. In humans, pimagedine (aminoguanidine, Synvista Therapeutics, New Jersey) has been evaluated in a phase II trial in which 690 patients with T1D were randomised to receive twice daily dosing with placebo, pimagedine 150 mg, or pimagedine 300 mg for 2–4 years. The primary end point was the time to doubling of serum creatinine, while the secondary end points included evaluations of proteinuria, kidney function, and retinopathy. In terms of DR, fewer pimagedine-treated patients experienced a three-step or greater progression of retinopathy[102].

Carbonyl trapping compounds are not only restricted to aminoguanidine. Others include ALT-946 and OPB-9195. Although yet to be tested in DR, both compounds confer protection in experimental models of diabetic nephropathy[103,104]. Intervention strategies have also been developed to inhibit other steps in the AGE pathway. Pyridoxamine is a post-Amadori product inhibitor through its free radical scavenging abilities[105]. In diabetic rat retina, pyridoxamine has been shown to reduce acellular capillary formation and the accumulation of the immunoreactive AGE carboxymethyllysine[98]. From a clinical perspective, tolerability of pyridoxamine by T1D and T2D with overt nephropathy has recently been reported[106]. Using pyridoxamine as a lead compound for rational drug design, Khalifah and colleagues reported that the compound BST-4997 is more effective at Amadori product inhibition[107], although to date this has not been studied *in vivo*.

Another AGE intervention strategy involves the breaking of protein cross-links that occur in the latter stages of the AGE reactions. The rationale is that once the protein cross-links are reversed, function may be restored to the targeted protein. Cross-link breakers include ALT-711 and LR-90, which have been shown to reduce pathological aspects of experimental diabetic nephropathy[108,109] and also operate in an anti-inflammatory capacity[110]. ALT-711 has shown some encouraging results in human studies, with its ability to improve diastolic function in patients with stable diastolic heart failure and patients with vascular stiffening[111,112]. Whether cross-link breakers have a therapeutic benefit in DR remains to be explored. Finally, after the discovery of RAGE, interventional approaches have been developed that target AGE-mediated signalling through this receptor. Soluble RAGE (sRAGE) is a recombinant soluble form of RAGE encoding the extracellular ligand binding domain of RAGE[113], which captures and eliminates circulating AGEs. sRAGE suppresses diabetic atherosclerosis pathology[114], and recent findings indicate that sRAGE can reduce both vascular leakage and inflammation in the retina of diabetic mice[96].

## *Vascular Endothelial Growth Factor*

Vascular endothelial growth factor-A (VEGF-A) is a key player in the pathogenesis of retinal pathology that characterises DR, age-related macular degeneration (ARMD), and retinopathy of prematurity[115,116,117]. This is due to VEGF-A's diverse actions on the retinal vasculature, which include the ability to stimulate neovascularisation, attract bone marrow–derived endothelial cell precursors, influence the survival of endothelial cells through inhibition of apoptosis, promote vascular leakage and inflammation, and possibly protect neurons from injury[116,118,119,120,121,122]. VEGF-A is the prototype member of a gene family that includes placenta growth factor, VEGF-B, VEGF-C, VEGF-D, and the orf-virus–encoded VEGF-E[123]. VEGF-A mRNA transcription leads to the production of at least four principal isoforms containing 121, 165, 189, and 206 amino acids, respectively, with $VEGF_{165}$ the predominant isoform[123]. In recent times, therapies targeting either one or all of these isoforms have been tested in human trials. Three major anti-VEGF agents have been most extensively studied: pegaptanib sodium (Macugen, Pfizer, New York and Eyetech, New York), ranibizumab (Lucentis, Genentech, South San Francisco, California), and bevacizumab (Avastin, Genentech, South San Francisco, California).

- Pegaptanib (Macugen) — Pegaptanib is a pegylated oligonucleotide aptamer against $VEGF_{165}$ and the longer isoforms. It is currently the only aptamer that has achieved the U.S. Food and Drug Administration approval for use in human disease. Two concurrent, identically designed, large trials have studied pegaptanib in patients with choroidal neovascularisation. In the VEGF Inhibition Study in Ocular Neovascularisation (VISION) trials[124], pegaptanib was given intravitreously every 6 weeks for 48 weeks to patients with wet ARMD, comprising a total of nine treatments. The combined findings of VISION indicated that pegaptanib reduced vision loss by approximately 50% in the first year and stabilised vision in the second year[124]. Adverse effects were mild and attributable to the injection protocol rather than pegaptanib. These included traumatic lens injury (0.7% of cases), retinal detachment (0.6% of patients), and endophthalmitis (1.3% of cases). The safety of pegaptanib past the 2-year study period is not known. Recently, pegaptanib has been studied in DR. In a phase II, randomised, double-masked, multicentre, dose-ranging, controlled clinical trial of 172 patients, intravitreous pegaptanib (0.2, 1, and 3 mg) was administered every 6 or 12 weeks with additional injections or focal photocoagulation as required for a further 18 weeks. Pegaptanib (3 mg) improved vision, decreased DMO, and there was an approximate 50% reduction in the need for laser therapy[125]. In addition to benefits for DMO, of 16 people who had retinal neovascularisation at baseline, most subjects assigned to pegaptanib showed regression of neovascularisation by week 36[126].
- Ranibizumab (Lucentis) — Ranibizumab is a recombinant, humanised, monoclonal antibody containing the antigen-binding sequence capable of binding and inhibiting all isoforms of VEGF-A by preventing normal ligation to their receptors [VEGF receptor 1(VEGF-R1) and VEGF-R2]. Ranibizumab has been evaluated in a multicentre, 2-year, double blind, sham-controlled study of ARMD (MARINA trial, the Minimally classic/occult trial of the Anti-VEGF antibody Ranibizumab in the treatment of Neovascular ARMD; and the ANCHOR trial, the Anti-VEGF Antibody for the Treatment of Predominantly Classic Choroidal Neovascularisation in ARMD). Patients received 24 monthly intravitreal injections of ranibizumab (either 0.3 or 0.5 mg) or sham injections. Ranibizumab prevented vision loss and improved mean visual acuity, with low rates of serious adverse events in patients with minimally classic or occult (with no classic lesions) choroidal neovascularisation secondary to ARMD[127]. Ranibizumab has not been studied extensively in DR. In 2006, a small, 6-month study comprising 10 patients with chronic DMO reported that intraocular injections of 0.5 mg of ranibizumab at baseline and at 1, 2, 4, and 6 months significantly reduced foveal thickness and improved visual acuity[128,129]. Ongoing studies are required to determine whether ranibizumab has long-term benefit to patients with DMO[130].
- Bevacizumab (Avastin) — Bevacizumab is an anti-VEGF agent similar to ranibizumab. Both are derived from the same murine monoclonal IgG antibody against VEGF-A. The main difference at the molecular level between bevacizumab and ranibizumab is that bevacizumab is a larger molecule, which lengthens its systemic half-life from a few hours to 3 weeks, making it more suitable as a systemic chemotherapy agent. Bevacizumab is approved as an adjunct therapy for the treatment of disseminated colorectoral cancer, having significant clinical benefit, including increased survival for patients[131]. In a study of 79 patients with subfoveal neovascular ARMD, intravitreal bevacizumab (1.25 mg) was administered on a monthly basis until macular oedema, subretinal fluid, and/or retinal pigment epithelial detachment resolved. Results suggest that intravitreal bevacizumab is associated with an improvement in visual acuity, decreased retinal thickness, and a reduction in angiographic leakage[132]. In terms of DR, in 32 patients, intravitreal bevacizumab (6.2–1.25 mg) was associated with rapid regression of retinal and iris neovascularisation secondary to PDR[133]. Similar findings were reported in smaller studies of patients with DR[134,135]. Although bevacizumab is well tolerated by patients, there are some serious toxicities associated with systemic delivery. Bevacizumab has led to gastrointestinal perforations, proteinuria, bleeding, and the development of hypertension requiring medical intervention with antihypertensive medication. Arterial thromboembolic complications, including stroke, myocardial infarction, transient ischaemic attacks, and unstable angina have been noted[131,134]. Bevacizumab may be the preferred treatment for ocular neovascular pathologies over pegaptanib or ranibizumab, as in many parts of the world both pegaptanib and ranibizumab are not readily available, and their use may be prohibited by cost. Given this situation, bevacizumab's widespread clinical use, and to date no evidence of ocular inflammation or other adverse events in association with intravitreal injection[136], there are a number of ongoing trials. The U.S. National Eye Institute is comparing the efficacy of laser treatment, intravitreal bevacizumab, and combination therapy or sham injection on DMO[137]. Another trial is evaluating intravitreal bevacizumab on the development of DR in people with active progressive PDR[138].

Although recent evidence suggests that VEGF inhibition is beneficial in patients with cancer, rheumatoid arthritis, and vascular insufficiency[139,140], there are concerns about the potential for serious side effects when VEGF inhibitors are administered systemically. This is largely because VEGF is required for normal physiological processes, such as cyclic endometrial development, vascularisation of the placenta, and wound healing[123,141]. VEGF may also be an important contributor to reparative angiogenesis in cardiovascular and peripheral vascular disease. This feature of VEGF action has ramifications for patients with DR and ARMD who are at increased risk of these events[142,143,144,145]. The side effects of systemic administration could be avoided with local delivery of VEGF inhibitors, such as by intravitreal injection to patients with DR and ARMD. However, repeated intravitreal injection may lead to an increased risk of retinal infection, cataract, and glaucoma, as well as the possibility of systemic absorption. In addition, consideration may need to be given to whether inhibiting ocular VEGF influences normal retinal function. VEGF is constitutively expressed in normal eyes, and may contribute to the maintenance of vascular integrity and be neuroprotective[146,147]. The efficacy of VEGF inhibitors administered by either a systemic or local route to diabetic and ARMD patients will need to be assessed in terms of long-term safety.

## Corticosteroids

Corticosteroids are a class of agents that are well known for their anti-inflammatory properties, and more recently have been demonstrated to be antiangiogenic and reduce VEGF expression. In preclinical models of retinal disease including diabetes, corticosteroids (such as trimacinolone acetonide and dexamethasone) reduce vascular leakage, inflammation, retinal vascularisation, and the expression of inflammatory mediators (such as tumor necrosis factor-$\alpha$, P-selectin, and intracellular adhesion molecule-1)[148,149,150,151]. These findings have largely been translated into clinical studies. In a prospective study of 36 patients, increased vitreal VEGF was reduced by intravitreal triamcinolone acetate (ITVA), which also attenuated retinal neovascularisation[152]. ITVA (4 mg) has also been reported to reduce foveal thickness and improve visual acuity in patients with DMO. However, IVTA does have side effects, such as cataract, increases in intraocular pressure, and infection[153,154,155,156]. Gillies and colleagues have conducted the most substantial study of ITVA in DR[155]. In a 2-year study, 19 of 34 (56%) IVTA-treated eyes had a visual acuity improvement of five letters or more, compared with nine of 35 eyes (26%) treated with placebo. However, cataract (55% of eyes) and intraocular pressure increases (68% of eyes) did occur[155].

As dosing may be an issue, intravitreal and retinal implants have been developed to allow extended drug delivery. Surgically implanted intravitreal fluocinolone acetone (Retisert, Baush & Lomb Pharmaceuticals, Rochester, New York) was evaluated in 197 patients with DMO who were randomised to receive either the implant or standard care. At 3 years, 58% of patients with implants experienced a resolution of DMO compared to 30% of controls. As for intravitreal injection, side effects, such as cataract and glaucoma, occurred in eyes that had received the implant. Posurdex (Oculex, Allergan Inc., California) is a drug-delivery system in a sustained-release formulation for posterior-segment delivery of dexamethasone for 35 days[157]. A phase II clinical trial of patients with macular oedema due to a variety of causes, including DR, experienced improvements in visual acuity of two or more lines on the ETDRS scale. This was associated with a decrease in retinal thickness, fluorescein leakage, and a trend toward improvement in visual acuity that appeared to be dose dependent[157].

The Diabetic Retinopathy Clinical Research Network (DRCRnet) is currently conducting a phase III randomised clinical trial comparing focal photocoagulation to ITVA for DMO. However, a major concern, based on clinical observations with intravitreal corticosteroids, is that DMO will recur as the effect of the intravitreal drug wears off, requiring repetitive injections long term. Combining an intravitreal drug (trimacinolone or ranibizumab) with photocoagulation may result in both short-term benefit of the intravitreal drug (decreased retinal thickening and decreased fluid leakage) and the long-term reduction in fluid leakage as a result of photocoagulation. The U.S. National Eye Institute is currently conducting a study to determine the most beneficial treatment for DMO: laser alone, laser combined with ITVA, laser combined with an intravitreal injection of ranibizumab, or intravitreal injection of ranibizumab alone[158].

## *Growth Hormone/Somatostatin*

The initial association between growth hormone (GH) and DR came from studies in which pituitary ablation was linked to the remission of DR[159,160]. It is now known that the GH/insulin-like growth factor-I (IGF-I) axis is altered in diabetes[161,162,163] and increased in vitreal fluids of diabetic patients[161,162,163]. Experimental studies have shown that IGF-I overexpression leads to severe retinal vascular and glial abnormalities, including neovascularisation and retinal detachment (reviewed [55])[164]. Therapies targeting inhibition of the GH/IGF-I axis have included somatostatin analogues, such as octreotide. Somatostatin is a neuropeptide that inhibits the release of a number of hormones including GH[165]. Somatostatin analogues have potent antiproliferative and antiangiogenic properties inhibiting ocular neovascularisation in experimental models[166,167,168,169]. However, octreotide has been reported to have variable results in patients with DR. A small, 1-year study found that in patients with early PDR, octreotide had no effect on fluorescein vascular leakage[170]. In contrast, in a pilot study of patients with severe NPDR or early non-high-risk PDR conducted over 15 months or until disease progressed to high-risk PDR requiring laser surgery, the incidence of ocular disease progression was only 27% in patients treated with octreotide compared with 42% in patients with conventional diabetes management[170]. More recently, a randomised controlled trial of patients with NPDR reported that octreotide retarded the progression of DR and delayed the time to laser treatment[171]. Two randomised clinical trials currently assessing long-acting octreotide injection in DR[172,173] have reported inconclusive preliminary data with adverse effects, such as diarrhoea, cholelithiasis, and hypoglycaemic episodes[174].

GH inhibition has also been studied as a therapeutic target for DR. In a study of 25 patients with either T1D or T2D with PDR, the GH receptor antagonist pegvisomant was given by subcutaneous injection for a 12-week period. This was followed by a 12-week period when patients were observed off treatment. Despite reducing serum IGF-I, diabetic patients experienced no change in retinopathy progression[175]. In contrast, experimental studies have shown GH antagonism to be beneficial for retinal neovascularisation. In transgenic mice expressing a GH antagonist gene and subjected to hypoxic-induced retinal neovascularisation, blood vessel growth is reduced[176]. Similar findings were recently reported with an antisense oligonucleotide against the GH receptor[177].

## Aldose Reductase Inhibitors

In diabetes, the polyol pathway metabolises excessive glucose. Aldose reductase is a key enzyme of the polyol pathway and reduces glucose to sorbitol using NADPH as a cofactor. Excessive accumulation of intracellular sorbitol found in various tissues of diabetic animals and in cells cultured under high glucose conditions has been proposed to be an important factor for the pathogenesis of diabetic complications. This increase in intracellular sorbitol is viewed to cause osmotic damage to cells[178]. Findings in experimental animal models have provided evidence that aldose reductase is a pathogenic factor in DR. For example, genetic deletion of aldose reductase prevents early effects of diabetes on neural, glial, and vascular cells of the retina[179]. Similarly, aldose reductase inhibitors (ARI) diminish the prevalence of microaneurysms, basement membrane thickness, oxidative stress, VEGF protein overexpression, neuronal apoptosis, and gliosis in animals with DR[180,181,182,183]. However, ARIs have yielded only minor benefits in clinical studies, such as the Sorbinil Retinopathy trial[184,185]. There is a suggestion that this poor performance may reflect insufficient inhibition of the pathway in human tissues[186].

Currently there are new ARIs that show a greater potency than sorbinil. Recently, an ARI from an entirely new structural class was described and reported to prevent elevated urinary albumin secretion in diabetic rats. ARI-809 is highly selective inhibitor of aldose reductase and in a model of streptozotocin diabetes, it improved survival, inhibited cataract development, normalised retinal sorbitol and fructose, and protected the retina from neuronal apoptosis, increased glial reactivity, intracellular adhesion molecule-1 expression, and complement activation[186]. The efficacy of new ARIs has yet to be established in patients with DR.

## Antioxidants: Vitamin E

The production of reactive oxygen species can occur from a variety of hyperglycaemia-induced events, including glucose autoxidation, increased traffic through the polyol pathway, and protein glycation. Animal studies indicate that antioxidants, such as vitamin E, may improve retinal haemodynamic changes in diabetes[187]. An 8-month clinical trial randomised 36 T1D and nine nondiabetic people to receive either 1,800 IU vitamin E/day or placebo for 4 months, and subjects were then followed, after treatment cross-over, for a further 4 months[188]. Oral vitamin E treatment normalised retinal blood flow abnormalities without a concomitant change in glycaemic control[188]. Vitamin E therapy has not yet been studied in a randomised clinical trial of patients with DR.

## Other Potential Treatments

A number of other molecules and systems of drug delivery are emerging as potentially important for the treatment of DR, and are briefly summarised here (Table 1)[189]. ATG-3 is an antagonist to the nicotinic acetylcholine receptor pathway that may reduce vascular leakage and angiogenesis in DMO. The Juvenile Diabetes Research Foundation (New York) in partnership with CoMentis (South San Francisco, California) has recently announced a phase II trial to evaluate the safety and activity of ATG-3 in improving vision in patients with DMO. The trial is expected to begin in late 2007 and conclude in mid 2008.

Pigment epithelial derived growth factor (PEDF) is an antiangiogenic molecule, which in the retina is expressed in retinal pigment epithelial cells. PEDF appears to act as a natural antagonist to VEGF, being down-regulated by tissue hypoxia and inhibiting VEGF binding to VEGF-R2 in DR[190]. PEDF is antiangiogenic in models of ischaemic retinopathy[191], and reduces vascular leakage and inflammation in experimental DR[192]. An adenovirus vector to PEDF has been developed by GenVec (GenVec Inc. Gaithersburg, MD) and is being tested in a phase I trial for wet ARMD[193].

VEGF Trap (Regeneron Pharmaceuticals, Tarrytown, New York) is a fusion protein which combines ligand-binding elements taken from the extracellular domains of VEGF-R1 and VEGF-R2 fused to the Fc portion of IgG1. VEGF Trap was designed to bind to all isoforms of VEGF-A with equal affinity, as well as placental growth factor. Experimental evidence indicates that VEGF Trap is effective in reducing vascular leakage, and retinal and choroidal neovascularisation in experimental models[194,195]. VEGF Trap administered by intravitreal injection is currently being investigated in a clinical trial of wet ARMD[196].

Small interfering RNA (siRNA) directed against VEGF-R1 mRNA has been developed to investigate the role of VEGF-R1 in ocular neovascularisation (Sirna-027, Sirna Therapeutics, San Francisco, California). Sirna-027 administered by intravitreal injection reduced retinal and choroidal neovascularisation in mice[197]. A phase I study evaluating Sirna-027 in wet ARMD is currently under way[189].

Cell-based therapies may also be beneficial for DR. Endothelial precursor cells (EPCs) play an important role in the repair and maintenance of the vascular wall. Recent experimental studies indicate that EPCs from diabetic patients exhibit impaired migration[198], while CD34+ cells from healthy volunteers are able to attach and be assimilated into the retinal vascular of diabetic animals to repair injured blood vessels[199]. Of interest is that the reduced migratory capacity of EPCs from diabetic patients may be correctable with exogenous nitric oxide[198]. IGF binding protein-3 (IGFBP3) may also be involved with a recent report indicating that IGFBP3 causes rapid differentiation of EPCs and intravitreal IGFBP3 administration protects the developing vasculature from the vessel regression that occurs in mice with oxygen induced retinopathy[200].

## CONCLUSIONS

Strict metabolic and blood pressure control, and laser photocoagulation, remain the conventional management for DR. However, experimental and clinical studies conducted over the past decade have seen a broadening of our understanding about the nature of the pathogenic factors involved in DR. We are also beginning to appreciate how these factors affect not only the retinal vasculature, but also neuronal and glial cell populations. New therapeutic avenues are now being evaluated with ongoing clinical trials exploring the role of angiotensin II blockade, intravitreal steroid, and inhibitors of VEGF, PKC, GH, aldose reductase, AGEs, and the nicotinic acetylcholine receptor. In the near future, results from clinical trials may lead to the introduction of additional or combination treatments and a corresponding reduction in the frequency of visual loss and blindness due to DR.

## ACKNOWLEDGEMENTS

Jennifer Wilkinson-Berka is a National Health and Medical Research Council (NHMRC) of Australia Senior Research Fellow.
